# Rapid intracranial response to osimertinib, without radiotherapy, in nonsmall cell lung cancer patients harboring the EGFR T790M mutation: Two Case Reports

**DOI:** 10.1097/MD.0000000000006087

**Published:** 2017-02-10

**Authors:** Taro Koba, Takashi Kijima, Takayuki Takimoto, Haruhiko Hirata, Yujiro Naito, Masanari Hamaguchi, Tomoyuki Otsuka, Muneyoshi Kuroyama, Izumi Nagatomo, Yoshito Takeda, Hiroshi Kida, Atsushi Kumanogoh

**Affiliations:** a Department of Respiratory Medicine, Allergy and Rheumatic Diseases, Osaka University Graduate School of Medicine; b Department of Immunopathology, Immunology Frontier Research Center, Osaka University, Japan.

**Keywords:** brain metastasis, osimertinib, case report, epidermal growth factor receptor, nonsmall cell lung cancer, T790M

## Abstract

**Rationale:**

Most of nonsmall cell lung cancer (NSCLC) patients harboring epidermal growth factor receptor (*EGFR*) activating mutations eventually acquire resistance to the first EGFR-tyrosine kinase inhibitors (TKIs) therapy after varying periods of treatment. Of note, approximately one-third of those patients develop brain metastases, which deteriorate their quality of life and survival. The effect of systemic chemotherapy on brain metastases after acquisition of EGFR-TKI resistance is limited, and thus far, whole-brain radiation therapy, which may cause the harmful effect on neurocognitive functions, has been the only established therapeutic option for especially symptomatic brain metastases. Osimertinib is a third-generation oral, potent, and irreversible EGFR-TKI. It can bind to EGFRs with high affinity even when the *EGFR* T790M mutation exists in addition to the sensitizing mutations. Its clinical efficacy for NSCLC patients harboring the T790M mutation has already been shown; however, the evidence of osimertinib on brain metastases has not been documented well, especially in terms of the appropriate timing for treatment and its response evaluation.

**Patient concerns, Diagnoses, and Interventions:**

We experienced 2 NSCLC patients with the *EGFR* T790M mutation; a 67-year-old woman with symptomatic multiple brain metastases administered osimertinib as seventh-line chemotherapy, and a 76-year old man with an asymptomatic single brain metastasis administered osimertinib as fifth-line chemotherapy.

**Outcomes:**

These patients showed great response to osimertinib within 2 weeks without radiation therapy.

**Lessons:**

These are the first reports to reveal the rapid response of the brain metastases to osimertinib within 2 weeks. These cases suggest the possibility that preemptive administration of osimertinib may help patients to postpone or avoid radiation exposures. In addition, rapid reassessment of the effect of osimertinib on brain metastases could prevent patients from being too late to receive essential radiotherapy.

## 1 Introduction

The epidermal growth factor receptor (EGFR)-tyrosine kinase inhibitors (TKIs) such as the first-generation gefitinib and erlotinib and also the second-generation afatinib showed remarkable activity in nonsmall cell lung cancer (NSCLC) patients harboring *EGFR* activating mutations.^[1–4]^ However, most patients eventually acquire resistance to the first EGFR-TKI therapy after varying periods of treatment, and approximately one-third of patients develop brain metastases after acquisition of EGFR-TKI resistance.^[5,6]^ Progressions of brain metastases are generally poor prognostic factors in patients with NSCLC,^[7,8]^ and the effect of systemic chemotherapy on brain metastases after acquisition of EGFR-TKI resistance is limited. Thus, whole-brain radiation therapy (WBRT), which may cause harmful effect on neurocognitive functions,^[9,10]^ has been the only option for especially symptomatic multiple brain metastases.^[11]^

Osimertinib is the third-generation oral, potent, and irreversible EGFR-TKI. It can bind to EGFRs with high affinity even when the T790M mutation exists in addition to the EGFR-TKI-sensitizing mutations,^[12]^ and clinical efficacy has already been shown.^[13]^ However, the effect of osimertinib on the central nervous system (CNS) metastases has not been documented well in clinical situations, except for a few preclinical studies.^[14,15]^ Furthermore, the time schedules for response evaluation are still controversial.

We describe 2 patients who showed great response in symptomatic as well as asymptomatic brain metastases to osimertinib therapy within 2 weeks. Preemptive administration of osimertinib may help patients to postpone or avoid radiation exposures. In addition, rapid reassessment of the effect of osimertinib on brain metastases could prevent patients from being too late to receive essential radiotherapy.

## 2 Case reports

Written informed consent was obtained from both patients for the publication of this manuscript and accompanying images.

### 2.1 Case 1

**Figure 1:**
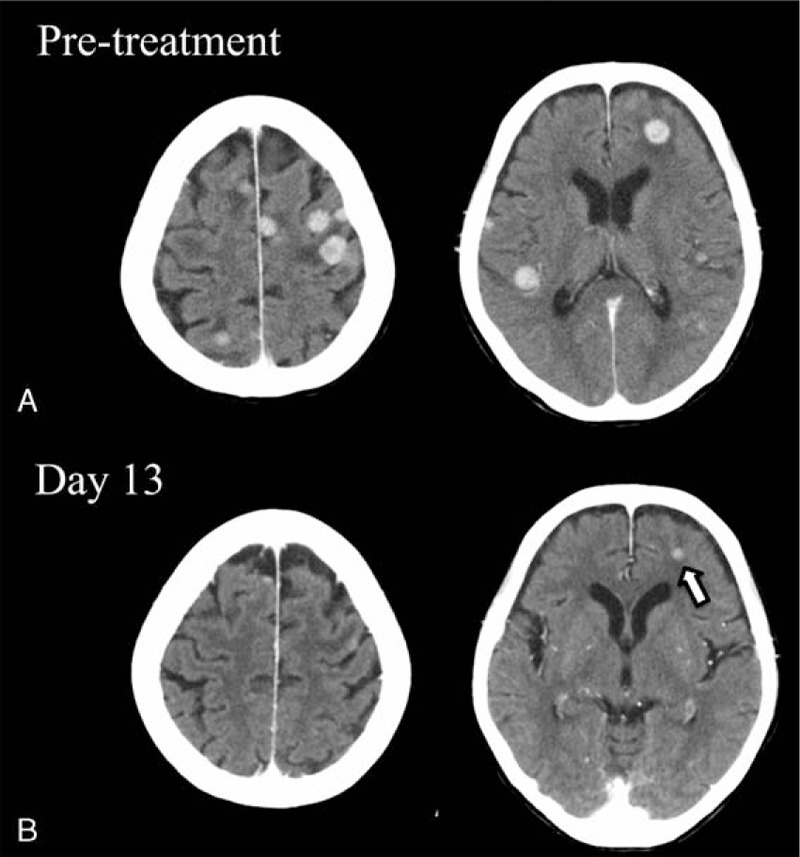
Contrast-enhanced axial brain CT images of case 1. Multiple metastases at baseline. Complete remission of brain metastases except for 1 (white allow) in the left frontal lobe after 13 days osimertinib administration. CT = computed tomography.

**Figure 2:**
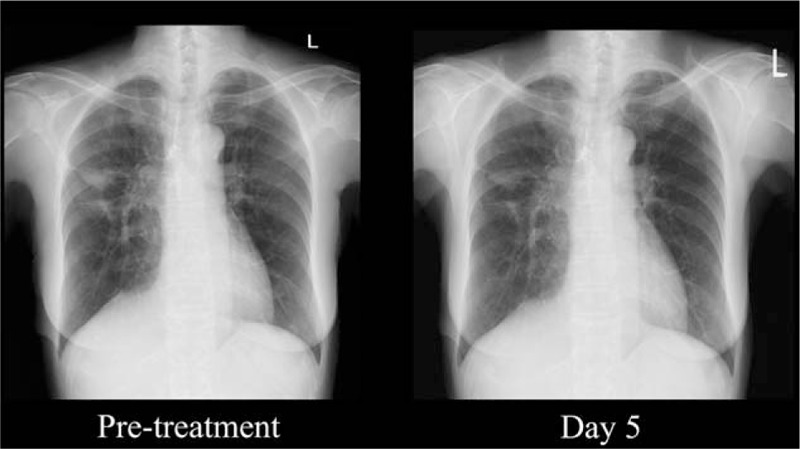
Chest x-rays of case 1. Shrinkage of the right pulmonary nodule after 5 days osimertinib administration.

A 67-year-old woman without a history of smoking, who had Behçet's disease, underwent right middle lobe resection of the lung because of an early stage of NSCLC 9 years ago. Multiple nodules emerged in both lungs on computed tomography (CT) images 7 years ago, which was recognized as a recurrence. An *EGFR* mutation analysis identified the *EGFR* L858R mutation in exon 21. Gefitinib was administered as first-line chemotherapy for 2 years and 11 months. After 3 lines of cytotoxic drug regimens (carboplatin plus pemetrexed, tegafur/gimeracil/oteracil plus bevacizumab, and docetaxel),erlotinib was administered as fifth-line chemotherapy and also as EGFR-TKI rechallenge, which failed in 3 and a half months. After 2 courses of vinorelbine administration as sixth-line chemotherapy, the patient complained of unbearable back pain, and spinal MRI examination detected multiple thoracolumbar bone metastases. Radiotherapy was conducted to control pain and prevent fractures. One month later, the patient complained of nausea and decrease of right grip strength. CT images revealed multiple contrast-enhanced nodules throughout the brain (Fig. 1A), and the symptoms were considered to be caused by brain metastases. After intravenous administration of corticosteroids and osmotic diuretics, the symptoms improved immediately. Bronchoscopy had been conducted as re-biopsy, detecting an additional mutation T790M in exon 20. As seventh-line chemotherapy, osimertinib (80 mg/day) was administered from the next day the brain nodules were detected, when the grade of Eastern Cooperative Oncology Group (ECOG) performance status (PS) was 3. On day 5 after administration of osimertinib was started, a chest x-ray revealed shrinkage of a right pulmonary nodule (Fig. 2). Grade 3 stomatitis and grade 2 diarrhea, classified by Common Terminology Criteria for Adverse Events version 4.0, emerged on day 9 and day 10, respectively. Osimertinib administration was discontinued on day 11. CT images on day 13 revealed complete remission of brain metastases, except for 1 lesion in the left frontal lobe (Fig. 1B). Nausea and decrease of right grip strength have not recurred, despite discontinuation of corticosteroids and osmotic diuretics. Her ECOG PS gradually improved to 2. In addition, serum alkaline phosphatase was elevated in parallel with tumor shrinkage, probably reflecting the healing process of bone metastases converting from osteolytic to osteoblastic state which is known as the flare phenomenon.^[16,17]^

### 2.2 Case 2

**Figure 3:**
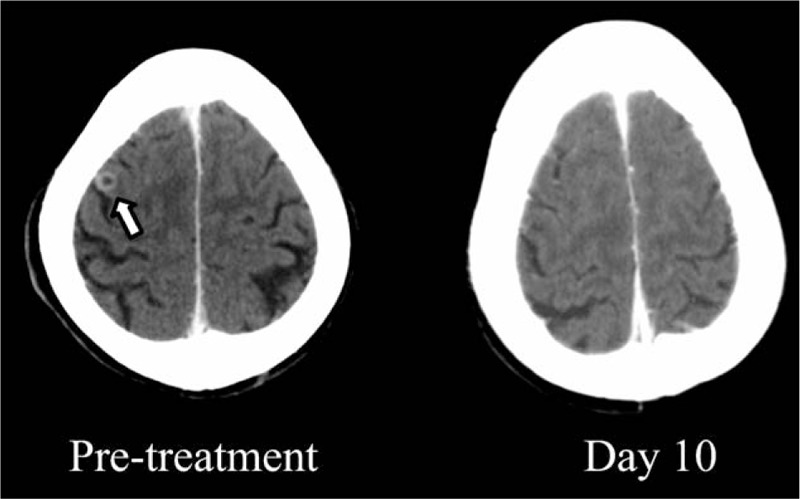
Contrast-enhanced axial brain CT images of case 2. Complete remission of a single brain metastasis (white allow) in the right parietal lobe after 10 days osimertinib administration.

**Figure 4:**
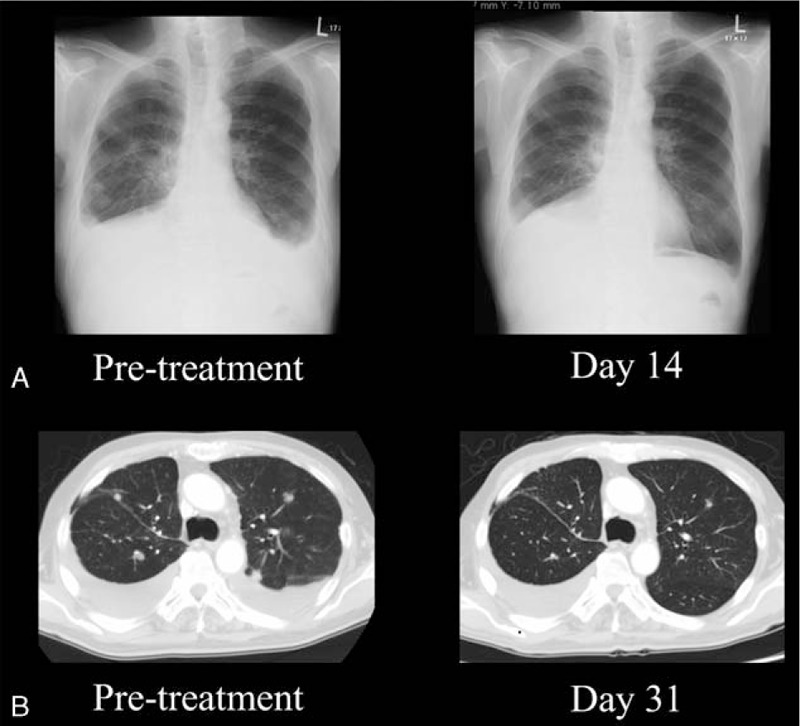
Chest x-rays and axial chest CT images of case 2. Decrease of pleural effusion and shrinkage of pulmonary nodules on chest x-rays after 14 days osimertinib administration. Shrinkage of multiple lung metastases and decrease of left-sided pleural effusion on chest CT images after 31 days osimertinib administration.

A 76-year old man, with a 22.5 pack-year smoking history, underwent right upper lobe resection of the lung because of an early stage of NSCLC 10 years ago. An *EGFR* mutation analysis identified the *EGFR* exon 19 deletion. Multiple bone metastases as recurrence emerged 6 and a half years ago. Gefitinib was administered as first-line chemotherapy for 4 and a half years. Afatinib was administered for 10 months as EGFR-TKI rechallenge after sandwiching 4 courses of carboplatin plus pemetrexed with bevacizumab regimen between the EGFR-TKIs. While fourth-line chemotherapy tegafur/gimeracil/oteracil was being administered, pleural effusion with right-sided dominance was seen. A thoracentesis was conducted and detected the additional mutation T790M in exon 20. Osimertinib was administered as fifth-line chemotherapy, when the ECOG PS grade was 2. On day 10 after administration of osimertinib was started, brain CT images revealed the remission of single brain metastasis in the right parietal lobe, which was asymptomatic (Fig. 3). On day 14, decrease of pleural effusion and shrinkage of pulmonary nodules were recognized apparently on a chest x-ray (Fig. 4A). After a month, chest CT images revealed shrinkage of multiple lung metastases and decrease of left-sided pleural effusion (Fig. 4 B). His ECOG PS gradually improved to 1, accompanied with the decrease of pleural effusion.

## 3 Discussion

We experienced 2 patients with NSCLC harboring the *EGFR* T790M mutation in addition to common activating mutations in whombrain metastases showed great response to the osimertinib treatment within 2 weeks. Although Ricciuti and coworkers reported 2 patients in whom brain metastases significantly responded to osimertinib treatment,^[18]^ this is the first report ever to reveal the rapid response of the brain metastases to osimertinib within 2 weeks.

Multiple brain metastases were symptomatic in the first case and WBRT was considered at first. However, disease progression was fast not only in the brain, but also in bones all over the body, and symptoms of the brain metastases were controllable by administration of corticosteroids and osmotic diuretics. We finally decided that the new systemic therapy with osimertinib should be given preference over the WBRT and succeeded in controlling brain metastases within 2 weeks.

Evidence for chemotherapy including osimertinib on brain metastases after acquisition of resistance to prior EGFR-TKI therapy is insufficient. Although there are some reports of effectiveness by dose escalation of EGFR-TKIs,^[19,20]^ pulsatile dosing of EGFR-TKIs^[21,22]^, and switch gefitinib to erlotinib.^[23,24]^ In the AURA phase II extension cohort (NCT01802632) and AURA2 phase II study (NCT02094261), total 162 of 411 patients (39%) had asymptomatic, stable brain metastases at entry.^[25]^ Objective response rate to osimertinib in patients with brain metastases was 56% (88/158; 95%CI 48, 64), comparable to 64% (154/239; 95%CI 58, 71) in patients without brain metastases.^[25]^ Thus, osimertinib may be effective enough in the treatment of NSCLC patients with brain metastases harboring the *EGFR* T790M mutation.

Overcoming blood brain barrier (BBB) is the key to have enough drug efficacy for CNS metastases, although parenchymal brain metastases may cause BBB disruptions and increase drug penetration to the brain.^[26,27]^ In a few preclinical models, osimertinib was suggested to have therapeutic potential in CNS metastases.^[14,15]^ Ballard and coworkers^[15]^ reported that osimertinib showed a good Kp_uu,brain_ value of 0.39 greater than other available EGFR-TKIs; Kp_uu,brain_ is well established as a good predictor of BBB permeability, with values greater than 0.3 indicative of good diffusion across the BBB.^[28]^

Compared to parenchymal brain metastases, leptomeningeal metastases appear to have distinct clinical courses and tumor biologies.^[29]^ Leptomeningeal metastases are especially life-threatening CNS complications, and EGFR-TKIs with high permeability to cerebrospinal fluid are essential. By update of ongoing phase I study for patients with leptomeningeal metastases from NSCLC called BLOOM (NCT02228369), osimertinib is expected to have enough efficacy by overcoming the BBB.^[30]^

WBRT has been the only option for symptomatic multiple brain metastases after acquisition of EGFR-TKI resistance.^[11]^ However, we should consider the potential harmful effect of radiotherapy, especially WBRT. Assessing impairment in neurocognitive function in patients treated with WBRT is difficult, because many variables including dysfunction caused by metastases themselves should be considered. Nonetheless, some studies supported these concerns with reasonable evidence,^[9,10]^ and delaying radiotherapy in favor of osimertinib may be a valuable option.

Although the dramatic responses to EGFR-TKIs are known to be observed in a few days in some cases, the time schedules for response evaluation are still controversial. Chang and coworkers^[31]^ reported that a very high percentage (21/29; 72.4%) of patients who achieved partial response to EGFR-TKI therapy demonstrated an early radiological response in 2 weeks. In addition, we reported that evaluation using ^[18]^ F-fluorodeoxyglucose uptake by positron emission tomography on day 2 after the initiation of gefitinib therapy is useful to predict the future clinical outcome of EGFR-TKI therapy at an early time point,^[32]^ although positron emission tomography is not appropriate to assess brain metastases generally.

Osimertinib is the only approved EGFR-TKI currently indicated for NSCLC patients harboring the *EGFR* T790M mutation. Patients acquire resistance to the osimertinib therapy after a median of 9.6 months of treatment.^[13]^ The *EGFR*C797S mutation has been reported as a major mechanism (6/15; 40%) of the resistance to osimertinib.^[33]^ Not surprisingly, brain metastases that had developed during the first EGFR-TKI treatment might respond once to osimertinib therapy but would become resistant. It is unknown whether we should recommend a fourth-generation EGFR-TKI that can overcome the resistance mediated by C797Sor local radiotherapy.

It is the best of all not to develop a brain metastasis in patients who are free of brain metastasis at diagnosis. There is no clinical evidence that supports osimertinib more efficacious in terms of postponing or preventing the development of brain metastases compared with first- and second-generation EGFR-TKIs. A phase III study FLAURA (NCT02296125) is ongoing comparing osimertinib with first-generation EGFR-TKIs as the first-line setting in patients with NSCLC harboring *EGFR-*activating mutations.^[34]^ This study could reveal if osimertinib will postpone or prevent disease progression by brain metastases compared with first-generation EGFR-TKIs.

In conclusion, osimertinib may be given priority over radiotherapy in terms of the therapeutic strategy for brain metastases of NSCLC harboring the *EGFR* T790M mutation, and the evaluation of treatment effect might be performed within 2 weeks. Further studies are needed to answer these clinical questions.
